# Intervention Using Video and Comic Books Targeting Nutritional Outcomes in Children and Adolescents: A Systematic Review

**DOI:** 10.1111/jhn.70362

**Published:** 2026-10-07

**Authors:** Ira Dwijayanti, Marian Abrafi Osei, Farah Nuriannisa, Luthfia Dewi, Arif Sabta Aji, Adi Lukas Kurniawan

**Affiliations:** ^1^ Department of Nutrition, Faculty of Health Universitas Nahdlatul Ulama Surabaya Surabaya Indonesia; ^2^ Transdisciplinary Research, Area (TRA) “Technology and Innovation for Sustainable Futures” and Center for Development Research (ZEF) Rheinische Friedrich‐Wilhelms University of Bonn Bonn Germany; ^3^ University Hospital, Kwame Nkrumah University of Science and Technology Kumasi Ghana; ^4^ Department of Nutrition Universitas Muhammadiyah Semarang Semarang Indonesia; ^5^ Research Center for Biomedical National Research and Innovation Agency Bogor Indonesia; ^6^ Heidelberg Institute of Global Health (HIGH) Medical Faculty and University Hospital, Heidelberg University Heidelberg Germany

**Keywords:** animated video, audiovisual aids, child and adolescent, comic books, dietary intake, nutrition literacy, nutritional status

## Abstract

**Introduction:**

Unhealthy eating behaviours and rising rates of undernutrition and obesity remain widespread among children and adolescents. Traditional education often fails to engage this population. In contrast, video and comic book formats offer accessible, engaging tools for nutrition education, but evidence on their effectiveness is inconsistent. This systematic review synthesises and critically appraises the evidence on video‐ and comic‐based interventions for improving nutritional outcomes in children and adolescents.

**Methods:**

In accordance with PRISMA guidelines a protocol was registered in PROSPERO (CRD420251138451). We systematically searched PubMed, Embase, and CENTRAL from January 2000 to October 2025 for randomised, cluster‐randomised, and non‐randomised intervention studies in participants aged ≤ 18 years using video‐ or comic‐based formats. Outcomes included nutritional status, dietary intake, food consumption, dietary patterns, and nutrition literacy. The risk of bias quality of the included studies were independently assessed by two reviewers.

**Results:**

Of 498 records, 18 studies met inclusion criteria—14 video‐based and 4 comic‐based interventions across school, digital, and clinical settings in Asia, Africa, Europe, North America, and Australia (sample sizes ranged from 57 to 6,640). Interventions consistently improved nutrition‐related knowledge, attitudes, and self‐efficacy. Effects on dietary behaviours, including fruit and vegetable consumption, were favourable, but rarely sustained at follow‐up. The effects of anthropometric factors on BMI and body composition were limited and inconsistent. Risk of bias ranged from some concerns to high or serious.

**Conclusion:**

Video‐ and comic‐based interventions effectively improve nutrition literacy among children and adolescents but show limited and inconsistent effects on dietary behaviours and anthropometric outcomes. Future interventions should move beyond information delivery and embed multimedia within multilevel strategies addressing behavioural, environmental, and parental determinants of diet.

## Summary

- Video and comic intervention formats consistently improved nutrition knowledge, attitudes and self‐efficacy in children and adolescents.
- Effects on dietary behaviours were favourable but rarely sustained at follow‐up, while effects on anthropometrics were limited and inconsistent.
- Durable behavioural change appeared more closely linked to repeated, reinforced exposure and to parental or environmental support than to programme length alone.
- Given the high risk of bias across studies, these media are best used within multilevel interventions rather than as standalone education.

## 1 Introduction

Optimal nutrition during childhood and adolescence is critical for healthy growth, cognitive development, and prevention of chronic diseases later in life [1]. However, despite global public health initiatives, unhealthy dietary behaviours—such as the frequent consumption of energy‐dense, nutrient‐poor foods and insufficient intake of fruits and vegetables—remain widespread among young people [2, 3]. This has led to an increase in the global prevalence of poor nutritional outcomes, including undernutrition, overweight and obesity [4]. A key component of addressing these challenges is effective health education that empowers young people to make informed decisions about their diet and lifestyle [5]. Traditional educational approaches frequently struggle to capture this demographic's attention, underscoring the need for innovative, participatory approaches. In recent years, digital and multimedia tools have gained popularity as promising options for health promotion due to their widespread accessibility and appeal to younger audiences [6, 7].

Educational interventions incorporating multimedia formats have gained prominence for their capacity to improve children's interest and enhance message retention [8, 9]. Videos, through their integration of visual, auditory, and narrative elements, can deliver complex health‐related information in accessible ways, appealing to diverse learning styles [10, 11]. Similarly, comic books leverage storytelling, visual cues, and relatable characters to convey health messages in an entertaining and informative way [12]. Both formats may serve as powerful tools for health promotion by bridging the gap between traditional nutrition education approaches and today's youth's communication preferences.

Despite the promise of these approaches, evidence on their effectiveness in shaping nutrition literacy, nutrition status, and dietary practices among children and adolescents remains scattered [13, 14, 15]. This systematic review aims to synthesise and critically appraise the existing evidence on the effectiveness of video and comic book interventions for improving nutritional outcomes among children and adolescents. We identified, screened, and analysed studies that have used these specific media to target a range of nutritional behaviours and outcomes, including knowledge, attitudes, dietary practices, and anthropometric measurements. By systematically reviewing the current literature, this study identified key intervention characteristics, assessed their impact, and highlighted research gaps to inform future public health initiatives and educational strategies.

## 2 Materials and Methods

### 2.1 Data Source and Search Strategy

This systematic review was conducted in accordance with the Preferred Reporting Items for Systematic Review and Meta‐Analysis (PRISMA) guidelines. A predefined review protocol was registered in PROSPERO (ID: CRD420251138451). As this study is based on aggregated data from previously published studies in open‐source databases, ethical approval was not required. We systematically searched the MEDLINE (via PubMed), Embase, and CENTRAL (via Cochrane Library) databases from 1 January 2000 to 31 October 2025. We identified and built our search strategy using the Participant, Exposure, Outcome, and Study design (PEOS) framework. The combination of medical subject headings (MESH) and free‐text words was first implemented in PubMed and then adapted to other databases. The full search strategy is available in Supplementary Table 1.

### 2.2 Eligibility Criteria

Eligible interventions comprised animated or non‐animated video‐based or comic book–based health and nutrition interventions, including both stand‐alone and multicomponent interventions that incorporated video or comics elements. Comparators included usual care, standard nutrition education, a non‐nutritional education delivered via video or comics, or alternative intervention approaches not involving video or comics. Studies were included if conducted in children and adolescents aged 18 years or younger. Studies that had overlapping age ranges were also considered. Studies were also eligible for inclusion if the mean or median fell within the defined age range. We also included peer‐reviewed studies published in English, with no restrictions on sample size or intervention duration. Primary studies of the following study designs were eligible for inclusion: randomised controlled trials, cluster‐randomised, and non‐randomised intervention trials, including quasi‐experimental studies and controlled before‐and‐after studies. Observational studies, studies without comparator groups, and non‐primary research (e.g., reviews, editorials, commentaries) were not eligible for inclusion. However, studies conducted in both high‐income countries and low‐ and middle‐income countries were eligible. Finally, the primary outcomes of interest included nutritional status (e.g., anthropometric indicators, anaemia, micronutrient and macronutrient deficiencies), dietary intake (including macro‐ and micronutrient intake), healthy and unhealthy food consumption, dietary patterns, and nutrition literacy (including knowledge, attitudes, intentions, and self‐efficacy). Secondary outcomes included the acceptability, feasibility, and appropriateness of the intervention.

### 2.3 Data Selection

All identified records were imported into Covidence (Veritas Health Innovation, Melbourne, Australia) for management and screening. Duplicate records were removed prior to screening. Titles and abstracts were independently screened by at least two reviewers, followed by full‐text screening of potentially eligible studies. Discrepancies between reviewers were resolved through discussion or, where necessary, consultation with a third reviewer.

### 2.4 Data Extraction

Data extraction was conducted independently by at least two reviewers using an extraction form developed in Covidence. Extracted information included study characteristics (title, first authors, year of publication, country, and study design), participant characteristics (age, sex, sample size), intervention details (description of the intervention, duration, frequency), comparison conditions, outcome measures, and summary of main findings with point estimates and measures of variance. Where necessary, the study authors were contacted up to two times to obtain missing or unclear data.

The risk of bias (RoB) quality of the included studies was independently assessed by two reviewers. Randomised controlled trials were evaluated using the Cochrane Risk of Bias 2 (RoB2) tool, including the version adapted for cluster‐randomised trials (RoB2 CRT). Non‐randomised studies were assessed using the Risk of Bias in Non‐randomised Studies of Interventions (ROBINS‐I) tool. Disagreements were resolved through discussion or consultation with a third reviewer.

### 2.5 Synthesis of Evidence

Given the anticipated heterogeneity in intervention types and outcome measures, a meta‐analysis was not conducted. Instead, a narrative synthesis was undertaken following the Synthesis Without Meta‐analysis (SWiM) reporting guidelines. Findings were summarised in both textual and tabular formats. Effect estimates for continuous outcomes were reported as (if applicable) mean, mean differences, or beta coefficients with 95% confidence intervals comparing the intervention with the comparison group. Meanwhile, dichotomous outcomes were reported as (if applicable) risk ratios, odds ratios, or prevalence rate ratios with corresponding 95% confidence intervals.

## 3 Results

The study selection process is presented in Figure 1. A total of 498 records were identified through database searching. After removal of 50 duplicates, 448 studies were screened based on title and abstract, of which 373 were excluded. Seventy‐five full‐text articles were assessed for eligibility, and 57 were further excluded. Ultimately, 18 studies met the inclusion criteria and were included in this review, of which 14 used videos as part of the intervention, and 4 used a comic as the intervention platform.

**Figure 1:**
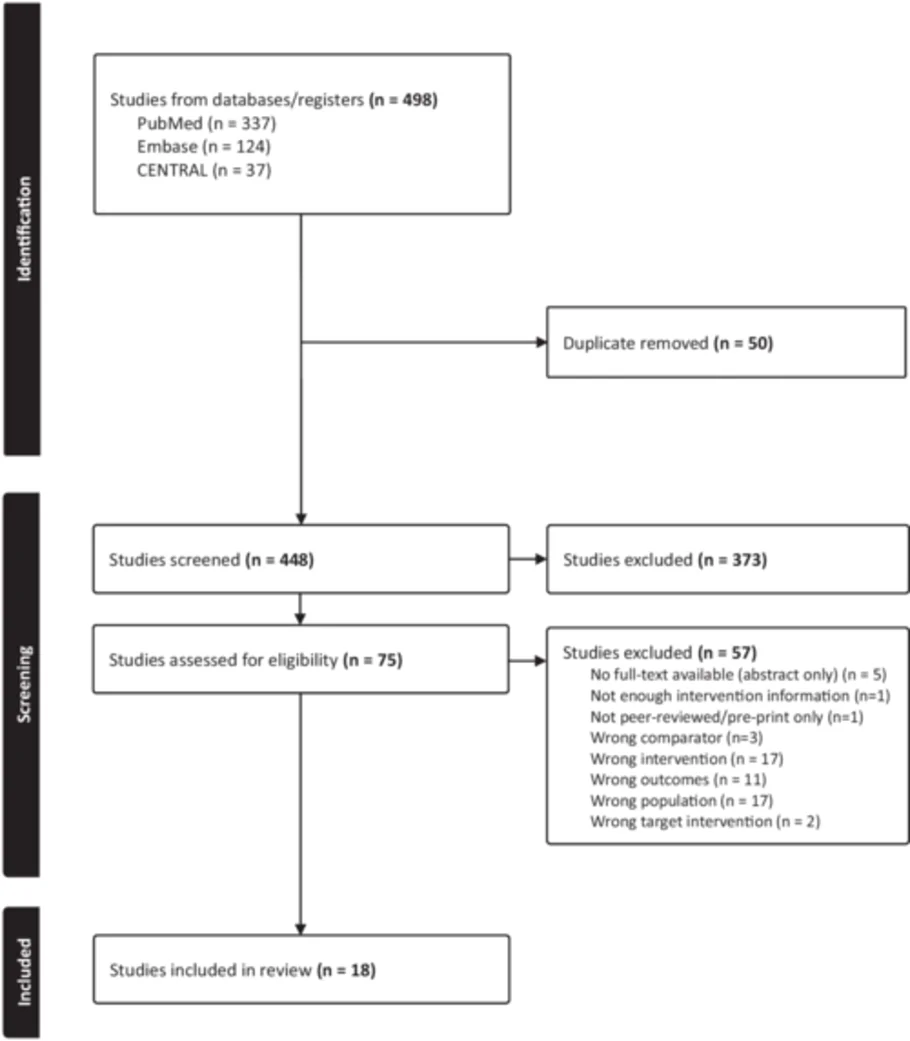
PRISMA systematic review flow chart.

### 3.1 Study Characteristics

The characteristics of the included studies are summarised in Tables 1 and 2. Of the 18 included studies, the majority were randomised controlled trials (RCTs), with sample sizes varying substantially, ranging from 57 to 6,640 participants. Geographically, studies were conducted across diverse settings, including Asia (Indonesia, Iran, Nepal, Taiwan, China, South Korea) [16, 17, 18, 23, 26, 28, 29], Australia [25], Africa (Cameroon) [20], Europe (Austria, the UK) [19, 21], and North America (the USA) [22, 24, 27, 30, 31, 32, 33]. Most interventions were implemented in school settings, followed by digital or mixed environments, and only one was conducted in a clinical setting [22].

**Table 1:** Characteristics of the included studies on video interventions targeting nutrition outcomes.

No	Author (year), country, design	Participants characteristics	Intervention	Comparator	Outcomes	Summary findings
1	Aisah (2022), Indonesia, QE[16]	**Sample:** 161, **Age:** 14–19 years old, **Gender:** Female, **Setting:** School	Animated 10‐min video with a topic on general anaemia information. **Duration:** NA. **Frequency:** three times. **Follow‐up:** 4 weeks post‐intervention	Received no intervention	Knowledge of Anaemia, self‐efficacy	The knowledge score for anaemia increased in the IG compared with the CG at follow‐up (mean: 102.0 vs. 97.0). The self‐efficacy score for obtaining iron tablets was higher in the IG than in the CG (mean: 15.5 vs. 15.0).
2	Asadabadi (2025), Iran, RCT[17]	**Sample:** 104, **Age:** 7–10 years old, **Gender:** All, **Setting:** Digital	13 exercise videos shared on the WhatsApp social network. **Duration:** 8 weeks. **Frequency:** three sessions per week, each 30 min. **Follow‐up:** immediately post‐intervention	Received no intervention	BMI (kg/m^2^)	After the intervention, the IG showed lower BMI (mean ± SD: 24.41 ± 1.73 vs. 25.58 ± 1.77) than the CG.
3	Bhandari (2025), Nepal, QE[18]	**Sample:** 167, **Age:** 12–18 years old, **Gender:** All, **Setting:** School	Nutrition education through interactive lectures: posters, educational videos, songs, and PowerPoints. **Duration:** 4 weeks. **Frequency:** 1 h session a day per week. **Follow‐up:** immediately post‐intervention	Regular school curriculum	BMI (kg/m^2^), nutrition knowledge, attitude, intention, and healthy eating behaviours	Mean scores for knowledge, attitude, and intention increased in the IG compared with the CG. No difference was observed at follow‐up in BMI (mean ± SD: 21.20 ± 4.77 vs. 22.52 ± 2.95, *p* = 0.05) or in the healthy eating behaviour score (mean ± SD: 4.26 ± 1.28 vs. 3.87 ± 1.23, *p* = 0.07) between the IG and the CG.
4	Binder (2020), Austria, Randomised parallel‐group experiment[19]	**Sample:** 161, **Age:** 6–10 years old, **Gender:** All, **Setting:** School	Three versions of a short cartoon movie, each ranging from 4:55 to 5:50 min in length. Gain‐framed group: the video featured arguments about eating fruit. Loss‐framed group: the video featured arguments about not eating fruit. **Duration:** not specified. **Frequency:** not specified. **Follow‐up:** not specified	The cartoon showed no food placement	Fruit intake	Children in the gain‐framed condition were more likely to choose fruit (ß=1.14, 95% CI: 0.10, 2.19) compared to children in the CG. No effect between the loss‐framed group and the CG.
5	Collishaw (2024), Cameroon, RCT[20]	**Sample:** 4306, **Age:** 10–12 years old, **Gender:** All, **Setting:** School	The five nutrition lessons cover nutrients, food groups, food safety, handwashing and hygiene, and wellness. For each lesson, an age‐appropriate French video was selected to complement the theme. Videos ranged from 1:42 to 3:38 min. **Duration:** 3 months. **Frequency:** not specified. **Follow‐up:** not specified	Traditional classroom instruction	Nutrition knowledge	Knowledge scores improved in both the control (non‐video pre vs. post mean ± SD: 2.56 ± 1.78 vs. 3.37 ± 2.27) and intervention (video pre vs. post mean ± SD: 2.60 ± 1.65 vs. 4.12 ± 2.45) schools. Video treatment increased the knowledge score by 0.45 (SE: 0.17).
6	Horne (2009), UK, RCT[21]	**Sample:** 435, **Age:** 4–11 years old, **Gender:** All, **Setting:** School	Children watched 6 video episodes (6 min each) featuring the heroic “Food Dudes”. Food Dude FV containers were provided to encourage parents to include FV in their children's lunchboxes. **Duration:** 16 days. **Frequency:** a day prior to lunch break. **Follow‐up:** 12 months post‐intervention	No animated video	FV consumption	At follow‐up, lunchbox FV consumption was significantly higher in the intervention school than in the control school (t = 3.7, *p* < 0.001). In the intervention school, consumption was significantly higher at follow‐up than at baseline (t = −5.5, *p* < 0.001), whereas in the control school, there was no significant difference.
7	Lewis (2023), USA, RCT[22]	**Sample:** 60, **Age:** 1–8 years old, **Gender:** All, **Setting:** Clinical	Multicomponent intervention: 1) water promotion toolkit, 2) a 5‐min educational video, 3) a mobile‐phone app called “Ready, Set, Gulp!” for tracking of SSB, fruit juice, and water intake, and 4) a series of 14 computerised interactive voice response phone calls. **Duration:** 6 months. **Frequency:** not specified. **Follow‐up:** 3 and 6 months	Received usual care	Consumption of SSB and fruit juice	Children in both the IG and CG decreased their reported frequency and total volume of SSB and fruit juice consumed over follow‐up. There were no significant between‐group differences in changes in servings (MD at 3‐months: −0.42, 95% CI: −1.01, 0.16; MD at 6‐months: −0.27, 95% CI: −0.97, 0.42) or volume of SSB and fruit juice for children at the 3‐ or 6‐month time points.
8	Li (2024), Taiwan, QE[23]	**Sample:** 65, **Age:** 9–10 years old, **Gender:** All, **Setting:** School	Four nutrition education modules on schoolchildren's nutritional issues, including Sugar Patrol, balanced diet, rainbow fruit and vegetables, and Smart Dine Out. Each course began with a 5‐min simulation video to stimulate students' learning motivation and guide them to connect with their life experiences. **Duration:** 1 month. **Frequency:** weekly for 40 min each. **Follow‐up:**	Health courses that were not related to nutritional education	Healthy eating knowledge, attitude, and behaviour	The intervention had no significant effect on the mean score for healthy eating knowledge (β = 2.45, *p* = 0.15). The intervention significantly affected attitudes toward healthy eating (β = 4.705, *p* = 0.048), but not healthy eating behaviour.
9	Nicklas (2017), USA, RCT[24]	**Sample:** 253, **Age:** mean age 4.4, **Gender:** All, **Setting:** School	Four 20‐min DVDs (videos) of theatre‐based puppet shows that aimed at persuading children to increase vegetable consumption. **Duration:** 4 weeks. **Frequency:** once a day. **Follow‐up:** 2 days post‐intervention	Received no intervention	Vegetable consumption	Children in the IG significantly increased vegetable consumption (*p* < 0.001) from baseline to follow‐up, whereas the CG showed no change. At follow‐up, the IG continued to have significantly higher intake of vegetable dishes (*p* = 0.022) than the CG. The intervention effect size was considered small (Cohen's d = 0.28).
10	O'Dean (2024), Australia, Cluster RCT[25]	**Sample:** 6640 (from 71 schools), **Age:** 11–14 years old, **Gender:** All, **Setting:** School	Health4Life: an e‐health program for multiple health behaviour changes to reduce increases in the six major behavioural risk factors for chronic disease: physical inactivity, poor diet, excessive recreational screen time, poor sleep, alcohol use, and tobacco use. The components are six 20‐min online cartoons, supplemented by internet‐based feedback, and a smartphone app. **Duration:** 6 weeks. **Frequency:** not specified. **Follow‐up:** immediately, 12‐ and 24 months post‐intervention	Received the usual health education	Discretionary food consumption and inadequate FV intake	The proportion of students who did not meet the fruit intake guidelines increased over time (24 months: control, 30.6% [95% CI: 28.7–32.6%]; intervention: 33.5% [95% CI: 31.7–35.5%]), but not that of those who did not meet the vegetable intake guidelines (24 months: control, 87.7% [95% CI: 86.3–89.0%]; intervention: 89.3% [95% CI: 88.0–90.4%]). The proportions reporting discretionary food risk decline slightly (24 months: control, 37.7% [95% CI: 35.7–39.8%]; intervention: 38.1% [95% CI: 36.1–40.1%]). The differences between Health4Life and CG at baseline and over 24 months were not significant for any outcomes.
11	Siswati (2022), Indonesia, QE[26]	**Sample:** 216, **Age:** 13 years old, **Gender:** All, **Setting:** Digital	Six online meetings covering topics on 1) anaemia and balanced nutrition guide, 2) adolescent roles toward zero stunting program, 3) reproductive health, 4) family planning, 5) juvenile delinquency, and 6) clean and healthy lifestyle. Students received the materials through videos following the online meeting. **Duration:** 3 weeks. **Frequency:** 60 min per session. **Follow‐up:** 1 and 3 weeks post‐intervention	Online meeting and received e‐leaflets	Knowledge and attitude toward the intergenerational cycle of malnutrition	Audiovisual (video) and e‑leaflet education increased the knowledge and attitudes of both groups. The IG showed a greater improvement in knowledge score than the CG (MD: 1.2 ± 1.1, *p* = 0.046). The increase in attitude scores was higher in the IG than in the CG (MD: 1.4 ± 0.9, *p* = 0.034).
12	Thompson (2023), USA, RCT[27]	**Sample:** 342, **Age:** 8–10 years old, **Gender:** Female, **Setting:** Digital	*Butterfly Girls and the Quest for Founder's Rock* (BFG) intervention to prevent obesity. The intervention, delivered online, consisted of 8 animated episodes promoting 5 servings of FV, 5 glasses of water, and 60 min of physical activity per day. Participants also set diet and physical activity goals, tracked and reported goal progress, and received feedback tailored to goal attainment. **Duration:** 3 months. **Frequency:** 8 online episodes. **Follow‐up:** 3‐ and 6 months post baseline	Comparison: Received only the animated stories. Waitlist control: Received no intervention	Healthy Eating Index (HEI) score	No significant effects were observed for the randomised group *x* time interaction or for group or time effects with any HEI score.
13	Yang (2017), South Korea, QE[28]	**Sample:** 768, **Age:** 9–13 years old, **Gender:** All, **Setting:** School	Educational dietary and exercise videos and the daily lunch menus were presented for 5–10 min a day via internet protocol television service for 1 academic year. Obese children require an ongoing vacation obesity care program during the 12‐week summer vacation. **Duration:** 1 academic year. **Frequency:** 5–10 min a day. **Follow‐up:** 1‐year post‐intervention	Received no intervention	BMI z‐score, weight, waist circumference (WC), % body fat, prevalence, incidence, and remission rates of overweight or obesity, and dietary habits	BMI classification changes did not differ significantly between the IG and the CG. The overweight/obesity incidence and remission rates did not differ statistically between the two groups. After 1 year, the IG showed a significant reduction in BMI z‐score compared with the CG (MD: −0.11, 95% CI: −0.16, −0.06, *p* < 0.001). A significant improvement in body composition with increased muscle mass (MD: 0.4, 95% CI: 0.1, 0.7, *p* = 0.005) and decreased % body fat (MD: −1.0, 95% CI: −1.5, −0.6, *p* < 0.001) was observed. WC and waist‐to‐hip ratio were significantly higher in the IG than in the CG (MD: 2.5, 95% CI: 1.9, 3.2 and MD: 0.014, 95% CI: 0.010, 0.018, respectively). The CG showed a greater increase in impulsively eating students after 1 year than the IG.
14	Zhang (2026), China, Cluster RCT[29]	**Sample:** 1085 (74 schools), **Age:** 8‐9 years old, **Gender:** All, **Setting:** School	*AppSalt and EduSaltS*: Twenty 5–10‐min video lessons on salt, diet, exercise, hygiene, and growth are pushed weekly, backed by books, posters, slides, online tools (FoodSwitch, quizzes, salt tracker, audio broadcast, pre‐ and post‐tests), and offline activities (poster displays, video screenings, parent meetings, canteen reforms). **Duration:** 10 months. **Frequency:** not specified. **Follow‐up:** 12 months and 24 months post‐intervention	Received no intervention	Change in salt intake and KAP	Both AppSalt and EduSaltS effectively reduced salt intake, but neither reached statistical significance. Pooling data from two trials showed that from baseline to the end of the study, salt intake increased in both CG and IG, with greater increases in the CG. Significant salt reduction through mHealth interventions: children decreased salt intake by 0.3 g/day (95% CI: 0.0, 0.7; *p* = 0.045). KAP scores increased significantly by 14.2 points (95% CI: 13.1, 15.4) in children.

Abbreviations: BMI, Body Mass Index; CG, Control group; CI, confidence interval; DVD, Digital Video Disc; FV, Fruits and Vegetables; IG, Intervention group; KAP, Knowledge, Attitude, Practices; MD, mean difference; RCT, Randomised controlled trial; SD, standard deviation; SSB, Sugar‐sweetened beverages; QE, Quasi‐experimental.

**Table 2:** Characteristics of the included studies on comics interventions targeting nutrition outcomes.

No	Author (year), country, design	Participants characteristics	Intervention	Comparator	Outcomes	Summary findings
1	Branscum (2013), USA, RCT[30]	**Sample:** 71, **Age:** 8–11 years old, **Gender:** All, **Setting:** School	*“Comic for health”*: Develop comic stories on the health issues covered during the lesson on 1) screen time, 2) consuming water and sugar‐free drinks instead of SSB, 3) exercise, and 4) consuming fruits and vegetables. **Duration:** not specified. **Frequency:** each lesson lasts for 30 min. **Follow‐up:** 3 months post‐intervention	Knowledge‐based: creating a comic not based on a health message	BMI for age percentile, FV consumption, and SSB consumption	No significant interaction (group‐by‐time) was found for BMI percentile or SSB consumption. Improvements over time (baseline to post‐test and baseline to follow‐up) were observed in both groups for FV consumption.
2	Ko (2025), USA, QE[31]	**Sample:** 653, **Age:** 8–12 years old, **Gender:** All, **Setting:** Multi‐settings	Multilevel intervention: individual, family, school, and community. Individual level: Two annual comic books for children with targeted messages about healthy eating and physical activity. Stories integrated the GO (a list of healthy foods), SLOW (foods to be taken in moderation), and WHOA (foods to avoid). **Duration:** 13 months. **Frequency:** annually (fall and spring). **Follow‐up:** 6 and 18 months post‐intervention	Not received a multilevel intervention, received two generic newsletters annually	BMI (kg/m^2^), BMI‐for‐age z‐score (BAZ), and BMI‐for‐age percentile.	The mean difference in BMI z‐score was 0.01 (95% CI: −0.04, 0.05; *p* = 0.78) among children in the intervention community relative to the comparison community. The mean difference in BMI z‐score at 6 and 18 months between the intervention and comparison community was −0.02 (95% CI: −0.05, 0.02; *p* = 0.31) and 0.03 (95% CI: −0.03, 0.09; *p* = 0.32), respectively. The mean differences in BMI‐for‐age percentile relative to the 95th percentile for the intervention community, compared with the comparison community, at 6‐ and 18‐month follow‐up were not significant.
3	Leung (2025), USA, RCT[32]	**Sample:** 89, **Age:** 9–12 years old, **Gender:** All, **Setting:** Digital	An interactive, web‐based 6‐chapter comic tool with pop‐up windows and prompted audio and visual effects, featuring health messages on fruit, vegetable, and water consumption.A weekly goal‐setting and assessment feature, plus reminder texts or emails. **Duration:** 6 weeks. **Frequency:** not specified. **Follow‐up:** 3 weeks post‐baseline (T2), 6 weeks post‐baseline (end of intervention) (T3), and 3 months post‐intervention (T4)	Received 6 web‐based newsletters	Self‐efficacy, intention, and attitudes related to fruit, vegetables, junk food, and sugary drinks. Frequency of fruit, vegetables, junk food, and sugary drinks consumption. BMI‐for‐age percentile.	The IG substantially increased fruit and vegetable intake, which was maintained at T4. Both groups reported reduced junk food consumption by T3, and the IG noted decreased sugary drink intake from T1 to T3; however, these improvements diminished at T4. The mean change between T1 and T4 in BMI‐for‐age percentile was small in both IG and CG (MD: –0.58, SD 5.91, and MD: –0.50, SD 11.49, respectively). Both groups improved FV self‐efficacy and intentions. Changes in beliefs and attitudes on sugary drinks and junk food were small and inconsistent. Effects were stronger in the IG at T3 but attenuated at T4, with only small between‐group differences.
4	Leung (2014), USA, RCT [33]	**Sample:** 57, **Age:** 8–15 years old, **Gender:** All, **Setting:** School	30‐page Manga comic titled *Fight for your right to fruit* with a health promotion message embedded in the narrative comic and a 1‐page non‐narrative to provide additional information about the benefits of fruit. **Duration:** 6 days. **Frequency:** 60 min. **Follow‐up:** immediately post‐intervention	Received a 5‐page newsletter and a word search puzzle on a non‐health‐related topic	Fruit snack selection, knowledge, and self‐efficacy related to fruit and fruit consumption.	Participants in the comic group were significantly more likely to choose a healthy snack than those in the CG (OR = 3.63, 95% CI: 1.09–12.1, *p* = 0.04). The Comic group showed significant within‐group changes in self‐efficacy (0.38 ± 0.92 vs. 0.06 ± 0.96, *p* = 0.04), but no significant change in knowledge (0.24 ± 0.65 vs. 0.20 ± 0.69, *p* = 0.07), compared with the CG.

Abbreviations: CG, control group; CI, confidence interval; BMI, Body Mass Index; FV, Fruits and vegetables; IG, Intervention group; MD, Mean difference; OR, odds ratio; QE, Quasi‐experimental; RCT, Randomised controlled trial; SD, standard deviation; SSB, Sugar‐sweetened beverages.

The interventions were categorised into two main types: video‐based interventions (*n* = 14) and comic‐based interventions (*n* = 4). Video interventions ranged from short, animated clips (3–10 min) to structured multi‐session educational programmes delivered over several weeks or months. Some interventions were standalone, while others were embedded within multicomponent programmes (e.g., mobile applications, classroom teaching, or behavioural tracking tools). Comic‐based interventions included narrative comics, interactive web‐based tools, and multilevel interventions incorporating family and community components. Additionally, the duration of interventions varied widely, from a few days to one academic year, and the follow‐up period ranged from immediate post‐intervention assessment to 24 months.

### 3.2 Risk of Bias Assessment

The RoB assessment is presented in Figure 2A‐C. Among the RCTs, studies were judged to have “some concern” and “high” levels, primarily due to deviations from the intended interventions and issues with outcome measurement. For cluster RCTs, one study demonstrated a high overall risk of bias [25], while another was judged low risk across most domains. Quasi‐experimental studies assessed using ROBINS‐I generally showed moderate to serious risk of bias, particularly due to confounding and participant selection biases.

**Figure 2:**
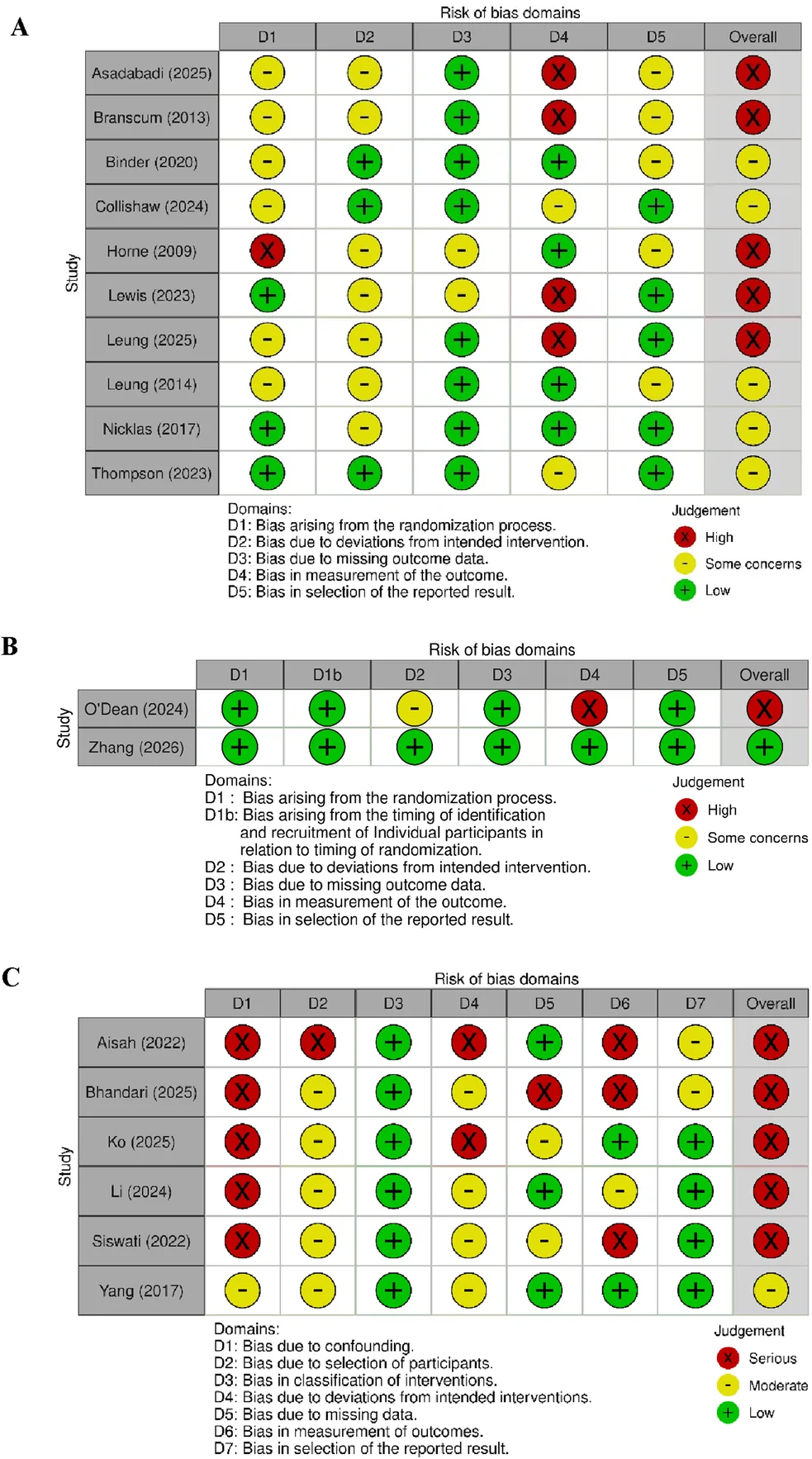
RoB assessment of included studies (A) randomised controlled trials, (B) cluster randomised controlled trials, and (C) non‐randomised controlled trials.

### 3.3 Effect of Interventions on Nutritional Outcomes

*Anthropometric outcomes*. Evidence on anthropometric outcomes was heterogeneous across studies (Tables 1, 2, and 3). Several video‐based interventions demonstrated modest improvements in BMI‐related indicators. For instance, an 8‐week video‐based exercise intervention significantly reduced BMI in children compared with controls [17]. Similarly, a long‐term school‐based intervention using daily educational videos reported significant reductions in BMI z‐scores and body fat percentage after 1 year [28]. However, several studies found no significant effects on BMI or BMI‐related indices, particularly in multicomponent or short‐term interventions [18, 30, 31]. Similarly, comic‐based interventions generally showed no significant impact on BMI or BMI‐for‐age percentiles [30, 31]. A limited number of studies assessed additional anthropometric outcomes. One long‐term video‐based intervention reported significant improvements in body composition, including increased muscle mass and reduced body fat percentage [28]. However, the same study observed increases in waist circumference and waist‐to‐hip ratio, indicating mixed effects across anthropometric measures [28].

**Table 3:** Summary of findings on the outcomes of included studies.

Outcomes	Author (year)																	
	Aisah(2013)[16]	Asadabadi (2025)[17]	Bhandari (2025)[18]	Binder (2020) [19]	Branscum (2013)[30]	Collishaw (2024)[20]	Horne (2009) [21]	Ko (2025) [31]	Leung (2014)[33]	Leung (2025)[32]	Lewis (2023)[22]	Li (2024) [23]	Nicklas (2017)[24]	O'Dean (2024)[25]	Siswati (2022) [26]	Thompson (2023) [27]	Yang (2017) [28]	Zhang (2026)[29]
BMI*																		
Fruit and vegetable consumption																		
Healthy eating behaviours^†^																		
Nutrition‐related attitude																		
Nutrition‐related intention																		
Nutrition‐related knowledge																		
Nutrition‐related self‐efficacy																		
Other anthropometric parameters ^‡^																		

*Note:* *Include BMI (kg/m^2^), BMI‐for‐age z‐score (BAZ), and BMI percentile. ^†^Include nutrition‐related behaviours, SSB consumption, healthy snacks, and other eating behaviours (e.g., junk food, discretionary food, salt intake, healthy eating index). ^‡^Include waist circumference, waist‐to‐hip ratio, body fat, weight, fat mass, and muscle mass. Green: significant intervention effect. Yellow: inconsistent results in one or more components of the outcomes, or a small intervention effect. Red: no intervention effect.

*Dietary behaviours*. Findings on dietary behaviours were variable but generally more favourable for specific targeted behaviours. Several video‐based interventions reported improvements in fruit and vegetable consumption. For example, children exposed to a cartoon‐based intervention were more likely to select fruit when presented with gain‐framed messages [19], while a school‐based intervention that used repeated exposure to video episodes significantly increased fruit and vegetable consumption at 12‐month follow‐up [21]. Similarly, a puppet‐based video intervention increased vegetable intake among preschool children [24]. However, not all interventions demonstrated significant effects. A multicomponent intervention combining video, mobile applications, and behavioural tracking did not significantly reduce SSBs or fruit juice consumption compared with usual care [22]. Likewise, a large cluster RCT evaluating a digital health intervention reported no significant improvements in fruit and vegetable intake or discretionary food consumption [25]. Comic‐based interventions showed promising effects on fruit and vegetable intake and on healthy snack selection (reduction in junk food intake), though these improvements were not consistently sustained over time [32].

*Nutrition knowledge, attitudes, and self‐efficacy*. Several video‐based interventions significantly increased participants' nutrition knowledge. For example, an animated video intervention improved knowledge and self‐efficacy related to anaemia among adolescent girls [16], while school‐based video‐supported lessons increased nutrition knowledge scores among children [20]. Similarly, multicomponent video‐based education programmes improved knowledge, attitudes, and intentions related to healthy eating [18]. Improvements in attitudes were also reported in some studies. A video‐based nutrition education intervention significantly improved attitudes towards healthy eating, although behavioural changes were not observed [23]. Likewise, audiovisual education, combined with e‐leaflets, improved both knowledge and attitude related to the intergenerational cycle of malnutrition [26]. Comic‐based interventions also demonstrated positive effects on psychosocial outcomes. An interactive comic intervention improved self‐efficacy, intentions, and attitudes related to healthy eating behaviours [32], while a narrative comic intervention increased self‐efficacy related to fruit consumption [33]. However, improvements in knowledge were not consistently observed [33]. Comic‐based interventions showed mixed effects. While reductions in junk food and sugary drink consumption were reported in the short term, these effects were not sustained at follow‐up [32]. Similarly, no significant intervention effect on behavioural changes was observed in other comic‐based interventions [30]. In addition, an mHealth‐supported video intervention targeting salt reduction demonstrated modest but statistically significant reductions in salt intake and improvements in knowledge, attitudes, and practices among children [29]. Overall, while some interventions showed beneficial effects on specific health indicators, findings were not consistent across studies.

Qualitative evidence was reported from two studies (Table 4) and highlighted high acceptability and engagement with both video and comic‐based interventions. Participants described the videos as engaging due to their visual appeal, storytelling, and interactive elements, which facilitated the understanding and retention of nutrition messages [32]. Similarly, comic‐based tools were perceived as enjoyable and game‐like, enhancing motivation and learning [24]. However, several barriers were identified, including limited attention span, time constraints, and competing activities. Suggestions for improvement included increasing parental involvement, incorporating interactive and sensory‐based learning approaches, and enhancing accessibility through digital platforms.

**Table 4:** Key themes and their summary results of qualitative evaluation.

Author (year)	Theme	Summary results
Leung (2025) [32]	Acceptability	The tool was acceptable, and very few reported any difficulties logging in or accessing content. Comics: Viewed as fun, engaging, and game‐like. Children reported learning about fruits, vegetables, and healthy behaviours through the storyline. Many children involved family members while reading the comic.
	Barriers	Barriers to goal setting: Time constraints and competing after‐school activities
	Feasibility	Goal‐setting tasks were generally perceived as manageable Text message reminders were preferred over emails due to convenience, clarity, and frequency of checking; most felt the number of messages was appropriate.
Nicklas (2017) [24]	Acceptability	Videos: High exposure, children repeatedly watched videos, strong appeal due to songs, and interactive characters. Improved understanding of vegetables (vitamins, energy, health benefits), and better differentiation between healthy and junk food. Increased willingness to try vegetables, children requested vegetables more often, and reinforced messages through repeated viewing.
	Barriers	Reduced attention due to hyperactivity, absenteeism, and behavioural issues.
	Suggestion	Introduce live demonstrations and puppet shows, enable sensory‐based learning (engaging all five senses), and include character visits or interactive experiences Increase parental involvement (e.g., joint sessions). Provide digital access (apps or web‐based platforms).

### 3.4 Theoretical‐Based Interventions

Many included studies were informed by theoretical frameworks, formative assessments, and, in some cases, co‐design with stakeholders. The “Together We STRIDE” programme was grounded in a Community‐Based Participatory Research framework and preceded a community‐wide needs assessment [31]. The intervention was subsequently co‐designed through active engagement with a Community Advisory Board comprising representatives from schools, social services, and law enforcement, who contributed to its planning and implementation. Similarly, the “web‐based comic” study was informed by multiple theoretical models, including the Health Belief Model, Social Cognitive Theory, and Narrative Transportation Theory [32]. The development process involved extensive formative research and usability testing, and the intervention was co‐designed by a multidisciplinary team comprising experts and members of the target population. The “Butterfly Girls” intervention also integrated Social Cognitive Theory and the Elaboration Likelihood Model, alongside community‐engaged research with children, parents, and community stakeholders to ensure cultural relevance [27]. Co‐design elements included developing a culturally sensitive narrative delivered through a professional playwright's storyline. Likewise, the motivational theatre intervention was informed by narrative transportation theory and Social Cognitive Theory and preceded by qualitative assessments with teachers and parents, whose input guided the development of characters and storylines [24]. The RESEPIN study combined audiovisual education theory with a needs assessment based on focus group discussions with public health stakeholders [26]. Other studies also integrated behavioural theories, including the Health Belief Model, the Family Ecological Model, the Transportation‐Imagery Model, and Social Cognitive Theory, with formative assessment through interviews and focus groups [16, 22, 33]. Several studies relied primarily on theoretical frameworks with limited needs assessment or co‐design. Interventions were commonly theory‐driven, with expert review or pre‐testing [18, 19, 30], while a few incorporated partial co‐design or user‐centred elements to enhance relevance and scalability [25, 29].

## 4 Discussion

This systematic review synthesised evidence on the effectiveness of video‐ and comic‐based interventions in improving nutritional outcomes among children and adolescents. Overall, these interventions were effective in improving nutrition‐related knowledge, attitudes, and self‐efficacy, but their effects on dietary behaviours and anthropometric outcomes were limited and inconsistent. Although some studies reported improvements in fruit and vegetable consumption and healthier food choices, these effects were not consistently sustained over time. Only a small number of studies found significant changes in BMI or body composition.

A key finding was the distinction between proximal cognitive outcomes and distal behavioural and anthropometric outcomes. Improvements in knowledge, attitudes, and self‐efficacy were among the most consistent results reported across multiple studies [18, 20, 26, 29]. Video‐based interventions improved nutrition knowledge among primary‐school children [20] and knowledge, attitudes, and intentions related to healthy eating [18, 26]. Comic‐based interventions also produced comparable psychosocial gains, improving self‐efficacy, intentions, and attitudes towards healthy eating [32] and self‐efficacy for fruit consumption [33]. Together, these findings indicate that both intervention formats effectively strengthen nutrition literacy and motivational determinants of behaviour. The qualitative outcomes within the review offer a plausible explanation, as participants found the video and comic materials engaging and enjoyable and reported that their visual, narrative, and interactive elements aided the understanding and retention of nutrition messages [24, 32]. This aligns with the wider evidence that multimedia formats can improve attention, comprehension, and retention [34] and that storytelling and relatable characters support observational learning and self‐efficacy; thereby increasing intentions to adopt healthier behaviours [35, 36].

However, these cognitive gains did not consistently translate into sustained behavioural change. A video‐based intervention improved attitudes towards healthy eating but produced no measurable change in behaviour [23], and a multi‐strategy programme incorporating educational videos improved knowledge, attitudes, and intentions yet showed no difference from the control condition in healthy‐eating behaviour or BMI at follow‐up [18]. Where behavioural improvements did occur, such as higher fruit and vegetable intake or healthier snack selection, these effects were often not maintained at follow‐up [24, 32, 33]. This reflects a common challenge in health promotion, namely that increased knowledge alone is often insufficient to produce lasting behaviour change [37]. Sustained change may also require environmental support, habit formation, reinforcement, and social influence [38], which many interventions in this review did not adequately address. Additionally, most included interventions primarily targeted individual‐level factors, with limited attention to broader contextual determinants [39].

The limited and inconsistent effects on anthropometric outcomes further underscore the complexity of achieving measurable health improvements. This is not surprising, as anthropometric change usually requires sustained behavioural modification over longer periods and is influenced by factors beyond diet, including physical activity, growth, and genetic predisposition [40]. The two interventions reporting favourable anthropometric change were both intensive or prolonged: a year‐long school programme delivering daily educational videos within a motivating environment reduced BMI z‐scores and body fat percentage [28], and an 8‐week video‐based exercise programme reduced BMI, plausibly through its physical‐activity component [17]. In contrast, short intervention and follow‐up periods may also have limited the ability to detect meaningful change. Shorter or predominantly educational interventions, such as the 4‐week multi‐strategy programme reported by Bhandari and colleagues, produced no significant change in BMI [18]. Even the year‐long programme showed mixed anthropometric effects, with concurrent increases in waist circumference and waist‐to‐hip ratio [28], which underlines the difficulty of achieving coherent anthropometric improvement through media‐based education alone. Intervention effectiveness also appeared to depend on design and implementation characteristics. Interventions that included repeated exposure, interactive components, or additional behavioural supports generally showed more favourable outcomes. For example, interventions combining video content with environmental cues or parental involvement were more likely to improve dietary behaviour [24, 27]. Moreover, repeated exposure to video episodes in the ‘Food Dudes’ programme increased fruit and vegetable consumption, which remained evident at 12‐month follow‐up [21]. In contrast, standalone educational interventions without reinforcement or contextual support were less likely to produce sustained change [41, 42, 43]. A standalone digital intervention produced no significant improvement in fruit and vegetable intakes [25], and encouraging parental involvement alongside simulation videos improved attitudes but not behaviour [23]. These within‐review contrasts suggest that multimedia tools may be most effective when embedded within broader multicomponent strategies.

Many included studies drew on theoretical frameworks, formative assessments, and, in some cases, stakeholder co‐design. These elements can strengthen intervention effectiveness and sustainability. Theoretical frameworks provide a structured foundation for intervention development, guiding strategy selection and ensuring alignment with established behaviour change mechanisms [44]. Thus, improving the clarity, reproducibility, and potential effectiveness of interventions. Pre‐defined needs assessments ensure that interventions are contextually relevant and tailored to the target population, while formative research helps identify user preferences, accessibility, and barriers [45, 46]. Additionally, co‐design approaches further improve acceptability, cultural relevance, and engagement by involving stakeholders in the development process, while also promoting long‐term adoption and sustainability through increased ownership [47]. However, even when interventions are rigorously developed using theoretical frameworks, formative research, and co‐design approaches, they often fail to achieve substantial effectiveness. The ‘Butterfly Girls’ programme [27], despite its basis in Social Cognitive Theory and community co‐design, found no significant improvement in diet quality. This suggests that while knowledge is essential, isolated behaviour change techniques such as goal setting are insufficient without broader self‐regulatory and reinforcement mechanisms [48]. Similarly, the ‘Together We STRIDE’ intervention [31] delivered much of its content at the individual and educational level and produced no anthropometric change, indicating that individual‐level education may have limited impact when environmental constraints, including parental control over food choices and broader socioeconomic or cultural factors, remain unchanged [49]. This may hinder behaviour change, particularly among children with limited autonomy. Finally, many interventions are of short duration, limiting their ability to support long‐term behaviour change, and may be affected by small sample sizes and measurement biases, particularly in self‐reported dietary outcomes, which are susceptible to recall and social‐desirability bias; accordingly, the study highlighted the need for more child‐friendly assessment methods [27]. These measurement limitations, together with biological preferences and cognitive demands, may hinder the application of nutrition knowledge into daily life [50].

Intervention effectiveness did not depend on programme duration and intensity. Two studies with the most pronounced anthropometric benefits were longer or more intensive [17, 28]. Yet, other extended interventions, including multilevel and multicomponent interventions, reported no anthropometric or dietary change [22, 31]. In contrast, several brief or single‐session interventions showed increased healthy dietary behaviour [19, 24, 33]. Notably, the most durable behavioural effect came not from the longest programme but from a brief but intensive, repeated‐exposure approach, which sustained at 12 months [21]. These indicate that repeated, reinforced exposure, rather than duration alone, distinguished sustained or durable effects from transient effects. Intervention effect patterns also showed no systematic variation by risk of bias, and reported benefits were not restricted to the more rigorously conducted studies. For example, studies judged to be at serious or high risk of bias frequently reported significant improvements in proximal outcomes (knowledge, attitudes, and self‐efficacy) [16, 18, 23, 26]. Conversely, null effects on dietary behaviours and anthropometric outcomes occurred both at moderate risk [27] and serious or high risk [25, 30, 31]. Meanwhile, the high‐risk study [21] and the only low‐risk study [29] both reported significant dietary behaviour effects. This contrast suggests that some reported benefits may partly reflect methodological limitations rather than true intervention effects, reinforcing the need for cautious interpretation of the more significant findings.

Our findings are consistent with previous literature on digital and school‐based nutrition interventions, which similarly report stronger effects on knowledge and attitudes than on behaviour or anthropometric outcomes [8]. This review extends the evidence by focusing on narrative and visual media formats, showing that while these approaches are engaging and acceptable, they are insufficient on their own to drive sustained behaviour change. The qualitative findings also suggest that time constraints, competing priorities, and limited environmental support may further reduce effectiveness in practice. This review has several strengths, including its focused scope, the inclusion of both quantitative and qualitative evidence, and the representation of diverse settings, thereby improving the relevance of the findings across contexts. However, several limitations exist. First, heterogeneity in study design, intervention characteristics, and outcome measures limited comparability and precluded meta‐analysis. Second, methodological limitations were common, with RoB assessments indicating moderate‐to‐serious concerns or high risk across several studies. Accordingly, the more favourable findings should be interpreted with caution. Third, many interventions were multicomponent, making it difficult to isolate the specific contribution of video or comic elements, and publication bias cannot be excluded. Fourth, several characteristics of the evidence further limit the generalizability of the findings. Sample sizes varied widely, meaning that smaller studies may have lacked statistical power, whereas a few large studies may have disproportionately influenced the overall evidence. Participants also spanned early childhood to late adolescence, across which nutritional needs, autonomy over food choices, and capacity to act on nutrition information differ markedly. Therefore, findings from one age group may not readily transfer to others. Lastly, our search was limited to three databases and to English‐language, peer‐reviewed publications, and did not include grey literature such as dissertations, standalone conference proceedings, or unpublished reports. Although CENTRAL captures many trial‐registry and conference records, these restrictions may have introduced publication and language bias.

These findings show video‐ and comic‐based interventions are promising tools for improving nutrition literacy and engagement, but their impact on behaviour and health outcomes is limited unless embedded within broader, multilevel strategies that address environmental, social, and behavioural determinants of diet. Integrating multimedia approaches with environmental modifications, parental involvement, and policy‐level interventions may enhance effectiveness. Future research should not simply add a single theory‐driven intervention but should explicitly target behaviour‐change mechanisms and embed a coordinated set of self‐regulatory strategies, such as goal setting, self‐monitoring, action planning, feedback, and reinforcement. Longer follow‐up periods and reinforced exposure to the interventions are needed to assess the sustainability of behavioural and anthropometric outcomes. Standardised outcome measures would also improve comparability and support future meta‐analysis. More high‐quality studies are needed in low‐ and middle‐income countries, where scalable and low‐cost interventions such as videos and comics may have substantial public health value.

## 5 Conclusion

In conclusion, video‐ and comic‐based interventions are effective in improving nutrition‐related knowledge and engagement among children and adolescents but show limited and inconsistent effects on dietary behaviours and anthropometric outcomes. Future interventions should move beyond information delivery and incorporate behavioural and environmental components to achieve sustained improvements in nutritional health.

## Author Contributions

A.L.K. and M.A.O. conceived the idea and developed the methods. I.D., M.A.O., and A.L.K. wrote the manuscript. I.D., F.N., L.D., and A.S.A. screened titles and abstracts, full texts, and extracted data. All authors reviewed, edited, and approved the final manuscript.

## Funding

The authors have nothing to report.

## Conflicts of Interest

The authors declare no conflicts of interest.

## Supporting information


Supporting File

## Data Availability

Not applicable, as all data utilised in this systematic review were derived from publicly available electronic databases and published peer‐reviewed literature. No primary data collection was conducted for this study.
