# Supplementary material for: Intervention Using Video and Comic Books Targeting Nutritional Outcomes in Children and Adolescents: A Systematic Review
Source: J Hum Nutr Diet. 2026 Oct 7;39(5):e70362. doi: 10.1111/jhn.70362 (PMC13646679; doi:10.1111/jhn.70362)
Supplement: Supplementary file 1 [file JHN-39-0-s001.docx]

**Supplementary Table 1.1. Search string for PubMed**

| **No** | **PEOS concept** | **Search strategy** |
| --- | --- | --- |
| #1 | **Participants** |  |
|  | Children and adolescents | (“Child”[Mesh] OR child*[tiab] OR "Child, Preschool"[Mesh] OR preschool*[tiab] OR “preschool child*”[tiab] OR "Infant"[Mesh] OR infant*[tiab] OR toddler*[tiab] OR under-five*[tiab] OR “under five child*”[tiab] OR “Adolescent"[Mesh] OR adolescent[tiab] OR adolescents[tiab] OR adolescence[tiab] OR teen[tiab] OR teens[tiab] OR teenage*[tiab]) |
| #2 | **Exposure** |  |
|  | Video and comic books | ("Video Recording"[Mesh] OR "Narration"[Mesh] OR video*[tiab] OR "education* video*"[tiab] OR film*[tiab] OR movie*[tiab] OR animation[tiab] OR cartoon*[tiab] OR “animated video*”[tiab] OR “short video*”[tiab] OR “short film*”[tiab] OR “Graphic Novels as Topic”[Mesh] OR "Books, Illustrated"[Mesh] OR comic*[tiab] OR “comic* book*”[tiab] OR “graphic* novel*”[tiab] OR storytelling [tiab]) |
| #3 | **Outcomes** |  |
|  | Nutrition status, nutrition intakes, nutrition literacy | (“diet”[Mesh] OR intake*[tiab] OR “diet* intake*”[tiab] OR “food intake*”[tiab] OR “nutritional status”[tiab] OR adequacy*[tiab] OR deficien*[tiab] OR micronutrient*[tiab] OR macronutrient*[tiab] OR “nutrient* adequacy*”[tiab] OR “nutrient* deficien*”[tiab] OR obesity*[tiab] OR obese[tiab] OR overweight[tiab] OR anemia[tiab] OR anaemia[tiab] OR underweight[tiab] OR stunting[tiab] OR stunted[tiab] OR malnutrition[tiab] OR malnourish*[tiab] OR wasted[tiab] OR wasting[tiab] OR undernutrition[tiab] OR “nutrition literacy”[tiab] OR "Health Literacy"[Mesh] OR “nutrition knowledge”[tiab] OR “nutrition attitude”[tiab] OR “nutrition self-efficacy”[tiab] OR “nutrition practice”[tiab]) |
| #4 | **Study design** | ("randomized controlled trial"[pt] OR "random allocation"[mesh] OR "cross-over studies"[mesh] OR "Controlled Before-After Studies"[Mesh] OR quasi experiment*[tiab] OR quasiexperiment*[tiab] OR “quasi-experiment*”[tiab) |

**Supplementary Table 1.2. Search string for Embase**

| **No** | **PEOS concept** | **Search strategy** |
| --- | --- | --- |
| #1 | **Participants** |  |
|  | Children and adolescents | ('child'/exp OR 'preschool child'/exp OR 'infant'/exp OR 'adolescent'/exp OR child*:ti,ab OR preschool*:ti,ab OR 'preschool child*':ti,ab OR infant*:ti,ab OR toddler*:ti,ab OR under-five*:ti,ab OR 'under five child*':ti,ab OR adolescent*:ti,ab OR adolescence:ti,ab OR teen*:ti,ab) |
| #2 | **Exposure** |  |
|  | Video and comic books | ('videorecording'/exp OR 'storytelling'/exp OR 'art'/exp OR 'medical illustration'/exp OR video*:ti,ab OR 'education* video*':ti,ab OR film*:ti,ab OR movie*:ti,ab OR animation*:ti,ab OR cartoon*:ti,ab OR 'animat* video*':ti,ab OR 'short video*':ti,ab OR 'short film*':ti,ab OR comic*:ti,ab OR 'comic* book*':ti,ab OR 'graphic* novel*':ti,ab OR storytelling:ti,ab) |
| #3 | **Outcomes** |  |
|  | Nutrition status, nutrition intakes, nutrition literacy | ('diet'/exp OR 'dietary intake'/exp OR intake*:ti,ab OR 'diet* intake*':ti,ab OR 'food intake*':ti,ab OR 'nutritional status':ti,ab OR adequacy*:ti,ab OR deficien*:ti,ab OR micronutrient*:ti,ab OR macronutrient*:ti,ab OR 'nutrient* adequacy*':ti,ab OR 'nutrient* deficien*':ti,ab OR obesity*:ti,ab OR obese:ti,ab OR overweight:ti,ab OR anemia:ti,ab OR anaemia:ti,ab OR underweight:ti,ab OR stunting:ti,ab OR stunted:ti,ab OR malnutrition:ti,ab OR malnourish*:ti,ab OR wasted:ti,ab OR wasting:ti,ab OR undernutrition:ti,ab OR 'nutrition literacy':ti,ab OR 'health literacy'/exp OR 'nutrition knowledge':ti,ab OR 'nutrition attitude':ti,ab OR 'nutrition self-efficacy':ti,ab OR 'nutrition practice':ti,ab) |
| #4 | **Study design** | ('randomized controlled trial':af OR 'randomization'/exp OR 'crossover procedure'/exp OR 'epidemiology'/exp OR quasi experiment*:ti,ab OR quasiexperiment*:ti,ab OR 'quasi-experiment*':ti,ab) |

**Supplementary Table 1.3. Search string for Cochrane**

| **No** | **PEOS concept** | **Search strategy** |
| --- | --- | --- |
| #1 | **Participants** |  |
|  | Children and adolescents | (mh “Child” OR mh “Child, Preschool” OR mh “Infant” OR mh “Adolescent”) OR (Child* OR preschool* OR preschool NEXT child* OR infant* OR toddler* OR under-five* OR under NEXT five NEXT child* OR adolescent* OR adolescence OR teen*):ti,ab,kw |
| #2 | **Exposure** |  |
|  | Video and comic books | (mh “videorecording” OR mh “art” OR mh “medical illustration”) OR  (video* OR education* NEXT video* OR film* OR movie* OR animation* OR cartoon* OR animat* NEXT video* OR short NEXT video* OR short NEXT film* OR comic* OR comic* NEXT book* OR graphic* NEXT novel* OR storytelling):ti,ab,kw |
| #3 | **Outcomes** |  |
|  | Nutrition status, nutrition intakes, nutrition literacy | (mh “Diet” OR mh “health literacy”) OR (intake* OR diet* NEXT intake* OR food NEXT intake* OR nutritional NEXT status OR adequacy* OR deficien* OR micronutrient* OR macronutrient* OR nutrient* NEXT adequacy* OR nutrient* NEXT deficien* OR obesity* OR obese OR overweight OR anemia OR anaemia OR underweight OR stunting OR stunted OR malnutrition OR malnourish* OR wasted OR wasting OR undernutrition OR nutrition NEXT literacy OR nutrition NEXT knowledge OR nutrition NEXT attitude OR nutrition NEXT self-efficacy OR nutrition NEXT practice):ti,ab,kw |
| #4 | **Study design** | (mh “Randomized Controlled Trial” OR mh “Random Allocation” OR mh “Cross-Over Studies” OR mh “Controlled Before-After Studies” OR mh “Non-Randomized Controlled Trials as Topic”) OR (quasi NEXT experiment* OR quasiexperiment*):ti,ab,kw |
